# New Drugs, Appliances, and Things Medical

**Published:** 1889-11-09

**Authors:** 


### NEW DRUGS, APPLIANCES AND THINGS MEDICAL.

[All preparations, appliances, novelties, etc., of which a notice is desired, should be sent to The Editor, The Lodge, Porchester Square, W.]

### TEMPERATURES—THE IMMISCH THERMOMETER.

The advance of modern medicine is in great measure due to improved methods of observation and research. The microscope, for instance, has opened up immense fields in the study of the changes in diseased tissue, and of the minute organisms that lie at the root of many of those processes. But in the actual bedside combat with disease, probably no single weapon has rendered more signal service than the thermometer. Its advantages are twofold—namely, in making out the nature and in foretelling the result of morbid action, while at the same time valuable hints for treatment are often afforded to the physician. As an aid to diagnosis no better example could be taken than the fevers. Compare, for example, the high initial temperature of scarlatina with the steadily rising chart of early enteric fever. As to prognosis, the severity of an attack can usually be determined by the thermometer; that a continuous high temperature in the last-mentioned fever—typhoid—is a danger signal, and a sharp and sudden rise during convalescence points to a relapse.

One of the greatest advances of modern times in medical treatment is reduction of raised temperatures, a rational method that has been largely founded on observations made with the thermometer. For many years the fact has been established that death follows a prolonged high temperature of about 104deg. F. and upwards. Different explanations have been offered, of which the most likely one appears to be coagulation of the substance of the muscle-fibres of the heart and other parts of the body. Be that as it may, the use of the thermometer has led to the great modern treatment of reduction of temperature, a scientific and exact method of checking the advances of raging fever. The physician has at disposal many means, of which the chief are baths, cold sponging, and such drugs as quinine, antipyrin, and antifebrin.

A word may be said about the curious cases of hyperpyrexia, in which persons are affected with high temperature extending over some time, without apparent cause or ill effects. In weighing such reports, however, it is always well to consider carefully certain points, such as accuracy of the thermometer used, presence of independent observation, and possibility of tampering with the instrument.

The first thermometers used were awkward, clumsy affairs, the length of a churchwarden pipe, and almost as brittle. Then it was found that a range confined to a few degrees above and below normal answered all practical purposes, and the instruments were shortened to a few inches. Improvements were made in the index, which was made self-registering, and at the same time kept from dropping into the bulb, where it would have been lost. Accuracy was ensured by careful testing and certification at the Kew Observatory. Flattening of the tube rendered the tiny index more visible, and made the thermometer less likely to roll off tables, a common cause of damage.

Many other improvements in detail have been made from time to time, but perhaps the most important embodiment is met with in the thermometer of Immisch, the only one yet made that is practically unbreakable. It has all the advantages mentioned in the other instruments, and has lately been perfected by the addition of a self-registering index. The saving in worry and expense effected by this little invention is remarkable. Shaped like a watch, it can be carried in the pocket, or fastened to a chain, and when in use can be detached and slipped into the armpit of the patient, which it fits exactly. Although its first cost is rather greater than the ordinary glass instrument, yet a few years will repay over and over again the extra outlay by the expenses saved in breakage of the latter. Nurses will find the Immisch thermometer well fitted for all purposes in and out of hospital, and every medical man should have one in his waistcoat pocket. *Crede experto.*

### SANTHA.

In an article last week dealing with "Santha," a new extract of tea, we described the tannin as combining to make an insoluble compound. The fact is the compound is soluble, and this feature of the new tea the manufacturers value very highly.

* *Lancet*, Oct. 12 1889.

